# p.Arg72Pro polymorphism of *P53* and breast cancer risk: a meta-analysis of case-control studies

**DOI:** 10.1186/s12881-020-01133-8

**Published:** 2020-10-19

**Authors:** Brehima Diakite, Yaya Kassogue, Guimogo Dolo, Jun Wang, Erin Neuschler, Oumar Kassogue, Mamadou L Keita, Cheick B Traore, Bakarou Kamate, Etienne Dembele, Sellama Nadifi, Robert L Murphy, Seydou Doumbia, Lifang Hou, Mamoudou Maiga

**Affiliations:** 1 Faculty of Medicine and Odontostomatology, 1805, Université des Sciences, des Techniques et des Technologies Sciences de Bamako (USTTB), Hamdallaye ACI, 2000 Bamako, Mali; 2 Teaching Hospital Center of Point G, 333 Bamako, Mali; 3 Preventive Medicine Department, Cancer Epidemiology and Prevention, Northwestern University, Chicago, IL 60611 USA; 4 Institute for Global Health, Northwestern University, IL60611, Chicago, USA; 5 Department of Radiology, College of Medicine, University of Illinois at Chicago, Chicago, IL 60612 USA; 6 Hassan II University Aïn chock, 20000 Casablanca,19, Rue Tarik Ibnou Ziad, Morocco

**Keywords:** *P53* gene, p.Arg/pro polymorphism, Breast cancer, Meta-analysis

## Abstract

**Background:**

The effect of the p.Arg72Pro variant of the *P53* gene on the risk of development ofbreast cancer remains variable in populations. However, the use ofstrategies such aspoolingage-matched controls with disease may provide a consistent meta-analysis. Our goal was to perform a meta-analysis in order to assess the association of p.Arg72Pro variant of *P53* gene with the risk of breast cancer.

**Methods:**

Databases such as PubMed, Genetics Medical Literature, Harvard University Library, Web of Science and Genesis Library were used to search articles. Case-control studies with age-matched on breast cancer havingevaluated the genotype frequencies of the *TP53 *p.Arg72Pro polymorphism were selected. The fixed and random effects (Mantel-Haenszel) were calculated using pooled odds ratio of 95% CI to determine the risk of disease. Inconsistency was calculated to determine heterogeneity among the studies. The publication bias was estimated using the funnel plot.

**Results:**

Twenty-one publications with 7841 cases and 8876 controls were evaluated in this meta-analysis. Overall, our results suggested that *TP53* p.Arg72Pro was associated with the risk of breast cancer for the dominant model (OR = 1.09, 95% CI = 1.02–1.16, *P* = 0.01) and the additive model (OR = 1.09, 95% CI = 1.01–1.17, *P* = 0.03), but not for the recessive model (OR = 1.07, 95% CI = 0.97–1.18, *P* = 0.19). According to the ethnic group analysis, *Pro* allele was associated with the risk of breast cancer in Caucasians for the dominant model and additive model (*P* = 0.02), and Africans for the recessive model and additive model (*P* = 0.03).

**Conclusions:**

This meta-analysis found a significant association between *TP53* p.Arg72Pro polymorphism and the risk of breast cancer. Individuals carrying at least one *Pro* allele *were* more likely to have breast cancer than individuals harboring the *A*rg allele.

## Background

Breast cancer is a multifactorial disease which constitutes a major public health problem [1]. In 2018, the World Health Organization reported that 2.09 million new cases of breast cancer were detected [1] compared to 1.38 million cases in 2008 [2]. It is the leading cause of death in women around the world. It should be noted that the incidence of breast cancer differs among different populations around the world [1]. Over the past decades, major advances have been made in understanding the pathology of breast cancer at the molecular level, including the involvement of certain genes associated with the development of the disease such as *BRCA1*, *BRCA2* and *P53* which produce tumor suppressor proteins and participate in damaged DNA repair [3–5]. *P53* plays a key role in the regulation of cell proliferation and apoptosis. The P53 protein is essential for maintaining the integrity of the cell and its components. In human cancers, mutated *P53* produces abnormal proteins that alter or inhibit transcriptional regulation [6]. As a result, the stress response, cell cycle as well as apoptosis are affected. Inactivation or mutation of *P53* gene would lead in linkage disequilibrium in the DNA sequence, which, associated with chromosomal aberrations induce the appearance of genomic instability and later the development of cancer [7, 8]. The molecular signature of human cancers shows that this gene is frequently observed in its mutated form [9]. *P53* has been mapped on chromosome *17p13* and contains 11 exons. Several single nucleotide polymorphisms (SNP) have been identified and the most studied variant is the substitution of Arginine by Proline at position 72 in exon 4. Studies carried out on different populations around the world have shown that this SNP is associated with the development of numerous diseases including cancers [10, 11]. It should be noted that, many association studies have examined the relationship between the SNP p.Arg72Pro of *P53* gene and the risk of breast cancer, however, the reports from these studies remain conflicting as some studies have shown that p.Arg72Pro is associated with the risk of breast cancer, while others found no association. Menzel et al. 2004 [12] and Akkiprik et al. 2009 [13] in their investigations showed a link between p.Arg72Pro and the risk of breast cancer. However, other authors who carried out a case-control study in which participants ages were not matched in a similar population, have concluded that p.Arg72Pro was not associated with the risk of breast cancer [14]. These different results with diverging conclusions can be explained by a very strong heterogeneity in allele and genotype distribution of p.Arg72Pro of the *P53* gene. This heterogeneity may be related not only to the geographic and ethnic origin [15–17] but also to the study design such as non-age-matched case-control studies. Based on these above observations, we hypothesized that the p.Arg72Pro polymorphism of *P53* gene may represent a potentially important genetic marker, contributing to breast cancer susceptibility in Caucasian, Asians and Africans. The present meta-analysis included only age-matched case-control studies in order to statistically decrease the heterogeneity between the studies, to qualitatively assess the effect of p.Arg72Pro on the risk of breast cancer. We have performed an independent two-stage meta-analysis; overall and sub-group analysis.

## Methods

### Literature search

The Pubmed Genetics Medical Literature Database, the Harvard University Library, and the Web of Science and Genesis Library were used to identify available articles published in English. The keywords “*P53*”, “p.Arg72Pro” and “polymorphism” or “mutation” or “gene” and “breast cancer”cited in the genetic association studies were used to detect and select scientific manuscripts in these databases. We also reviewed references cited in these studies to identify additional articles that were not identified by our research in the databases.

### Inclusion criteria

The inclusion criteria included: (1) published case-control studies as an original article to evaluate the association between p.Arg72Pro of the *P53* gene and the risk of breast cancer, (2) full manuscript available, (3) case-control study with age-matched, (4) distribution of genotype respecting Hardy-Weinberg equilibrium (HWE) in controls, (5) availability of the three genotypic frequencies (*Arg/Arg*, *Arg/Pro* and *Pro/Pro*) in the case and control groups. (6) Study no influencing the pooled odd ratio (OR) values. Three investigators independently evaluated each study to determine eligibility.

### Data extraction

The data were collected by an investigator and verified by a second investigator to reach consensus on all points. First author, year of publication, country, ethnicity of study population, sample size, age-matched, distribution of genotype andalleles, as well as the recalculation of HWE in controls were extracted from the eligible studies. A third reviewer made a contradictory assessment to reconcile the assumptions. The data of controls evaluated with p.Arg72Pro variant were included in this meta-analysis.

### Statistical analysis

Chi^2^ analysis with a significance level of *P* < 0.05 was used to evaluate whether p.Arg72Pro polymorphism distribution of the *P53* gene in controls fits HWE. The association between the p.Arg72Pro and the risk of breast cancer was evaluated by the Odd ratio (OR) of 95% CI. We evaluated the strength of association between the p.Arg72Pro polymorphism of *P53* gene and the risk of breast cancer using different genetic models, including the dominant (*Pro/Pro* + *Arg/Pro* vs. *Arg/Arg*), recessive (*Pro/Pro* vs. *Arg/Arg* + *Arg/Pro*) and the additive (*Pro* vs. *Arg*). Heterogeneity among the studies was assessed by I^2^ statistical test [18, 19]. If I^2^ > 50% (presence of heterogeneity), the random effects model was used to calculate the overall OR, otherwise in case of lack of heterogeneity, the fixed effects method was used. We also have examined the funnel plot to determine publication bias [20]. All statistical analyses were performed with Review Manager Software version 5.1.

## Results

### Characteristic of eligible studies

**Fig. 1:**
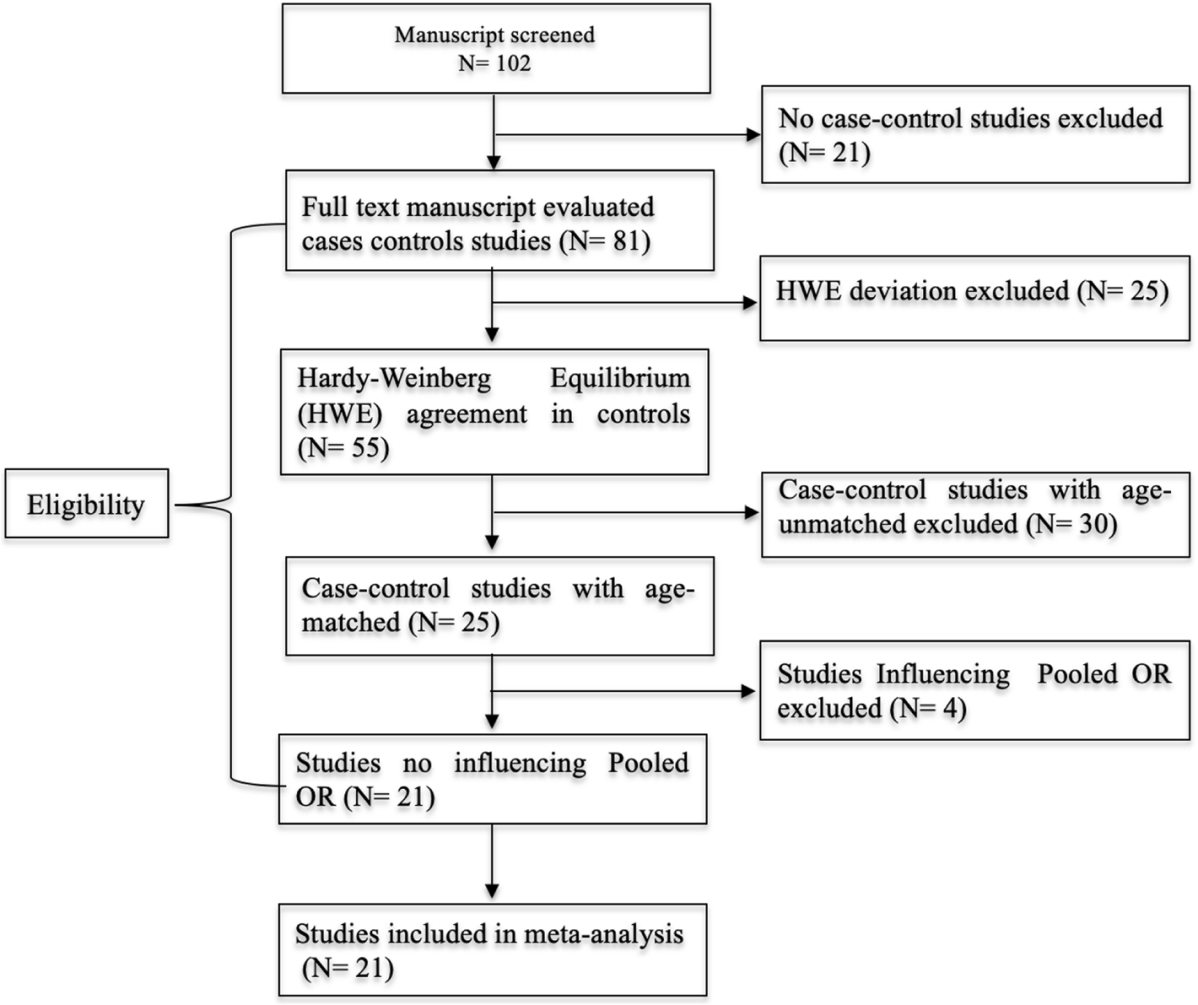
Flow diagram of the studies evaluated for meta-analysis

Figure 1 summarizes the process of selecting studies that the inclusion criteria. In sum, 21 eligible age-matched case-control studies were selected for the pooled OR analyses. Genotype distribution of the control population that met HWE was a minimum requirement for studies to be retained for the meta-analysis. Out of the 21 studies (7841cases and8876 controls), eleven were Caucasians [12–14, 23–28, 36, 37], nine were Asians [21, 29–35, 38] and one was African [22] (Table 1).

**Table 1:** Genotypes distribution of *TP53* p.Arg72Pro in breast cancer cases and controls

			Cases				Controls			
Authors	Ethnicity	N	***Arg/Arg***	***Arg/Pro***	***Pro/Pro***	N	***Arg/Arg***	***Arg/Pro***	***Pro/Pro***	HWE
Akkiprik et al. 2009 [13]	Caucasian	95	25	50	20	107	46	49	12	Yes
Alshatwi et al. 2012 [21]	Asian	100	22	52	26	100	32	51	17	Yes
Ayoubi et al. 2018 [22]	African	125	55	42	28	126	65	46	15	Yes
Buyru et al. 2003 [23]	Caucasian	115	64	39	12	63	26	28	9	Yes
Cherdyntseva et al. 2012 [24]	Caucasian	388	184	162	42	275	148	100	27	Yes
Costa et al. 2008 [25]	Caucasian	175	98	61	16	212	124	70	18	Yes
Cox et al. 2007 [26]	Caucasian	1477	804	569	104	2224	1255	838	131	Yes
Denisov et al. 2009 [27]	Caucasian	297	148	124	25	275	147	99	29	Yes
Ebner et al. 2010 [28]	Caucasian	263	138	108	17	254	137	103	14	Yes
Hossain et al. 2016 [29]	Asian	125	54	42	29	125	61	51	13	Yes
Isakova et al. 2017 [30]	Asian	117	57	50	10	102	53	36	13	Yes
Katiyar et al. 2003 [31]	Asian	77	20	51	6	41	9	24	8	Yes
krivokuca et al. 2014 [14]	Caucasian	155	87	58	10	114	62	45	7	Yes
Li et al. 2002 [32]	Asian	28	11	10	7	50	10	26	14	Yes
Ma et al. 2006 [33]	Asian	404	149	178	77	472	150	222	100	Yes
Menzel et al. 2004 [12]	Caucasian	302	158	114	30	475	275	170	30	Yes
Sharma et al. 2014 [34]	Asian	200	47	103	50	200	67	91	42	Yes
Song et al. 2009 [35]	Asian	1110	341	547	222	1097	355	514	228	Yes
Sprague et al. 2007 [36]	Caucasian	1653	909	644	100	1854	1021	704	129	yes
Wang-Gohrke et al. 2002 [37]	Caucasian	552	282	221	49	543	300	203	40	yes
Zhang et al. 2007 [38]	Asian	83	21	45	17	167	47	87	33	yes

*N* Number, *HWE*, Hardy-Weinberg Equilibrium

### Quantitative analysis

**Fig. 2:**
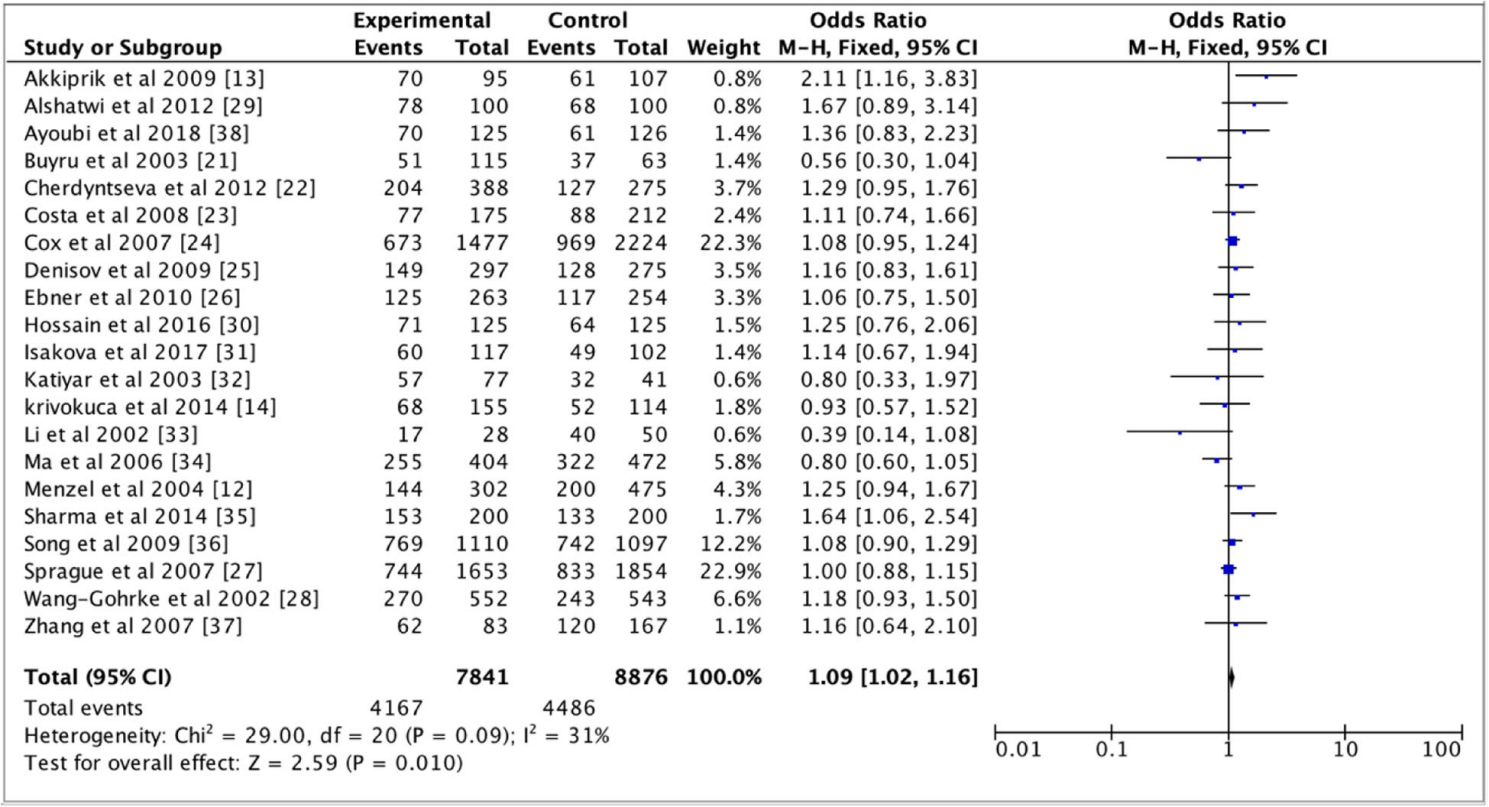
Forest plots of the association between breast cancer and *TP53* p.Arg72Pro polymorphism for the dominant model. The black diamond denotes the pooled OR; blue squares indicate the OR in each study with square sizes inversely proportional to the standard error of the OR; and horizontal lines represent the 95% CI

**Fig. 3:**
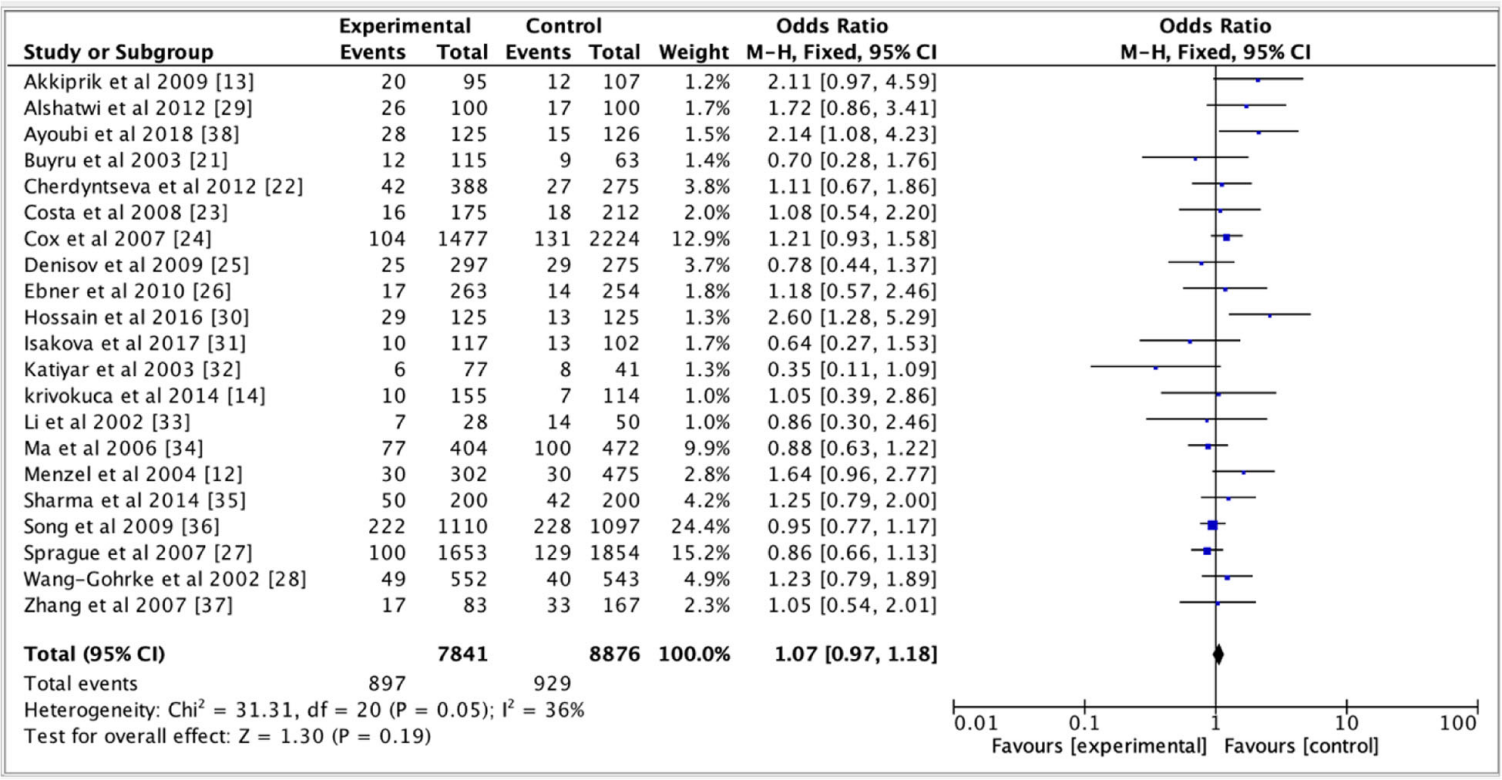
Forest plots of the association between breast cancer and *TP53* p.Arg72Pro polymorphism for the recessive model. The black diamond denotes the pooled OR; blue squares indicate the OR in each study with square sizes inversely proportional to the standard error of the OR; and horizontal lines represent the 95% CI

**Fig. 4:**
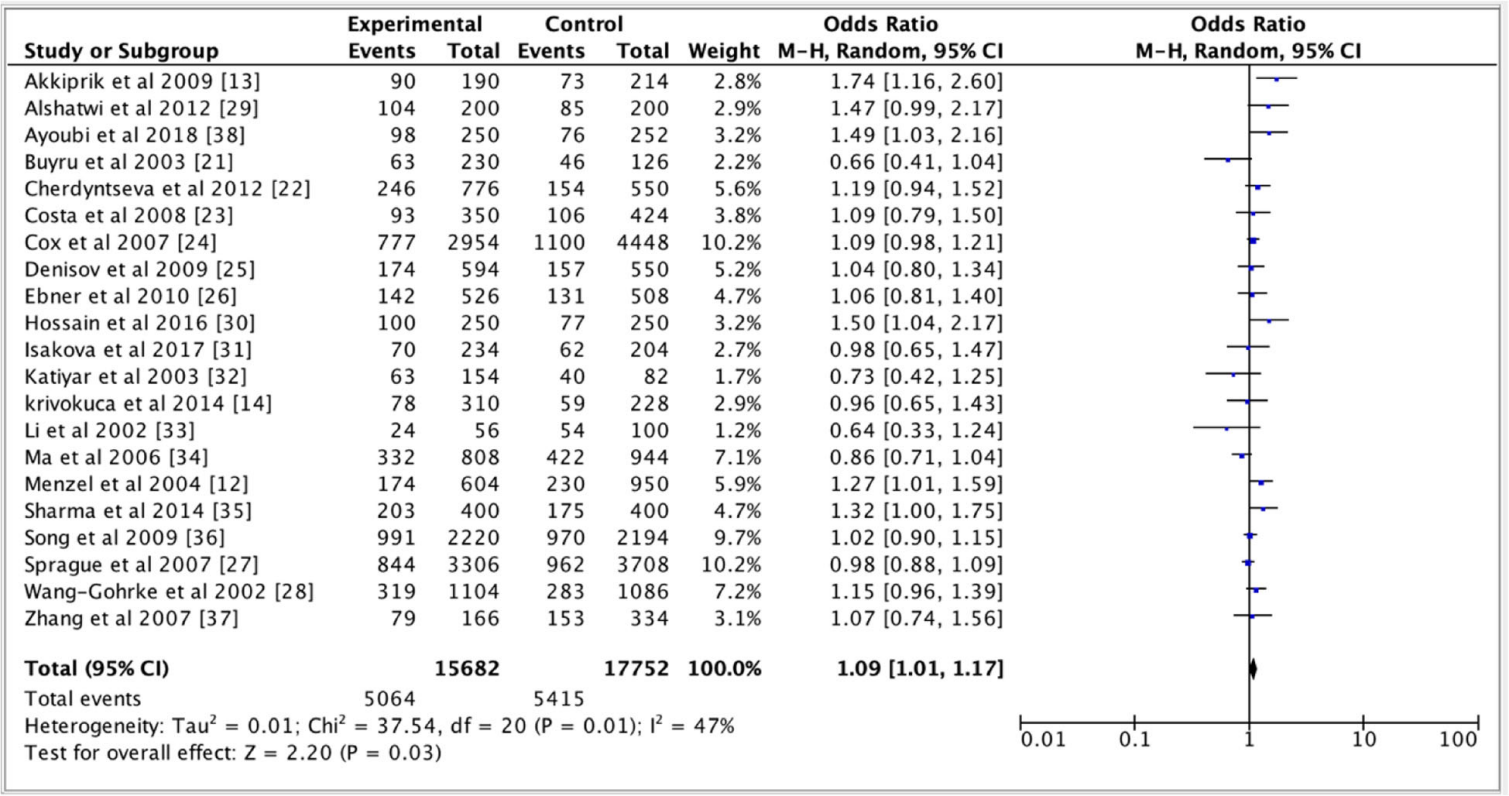
Forest plots of the association between breast cancer and *TP53* p.Arg72Pro polymorphism for the additive model. The black diamond denotes the pooled OR; blue squares indicate the OR in each study with square sizes inversely proportional to the standard error of the OR; and horizontal lines represent the 95% CI

**Fig. 5:**
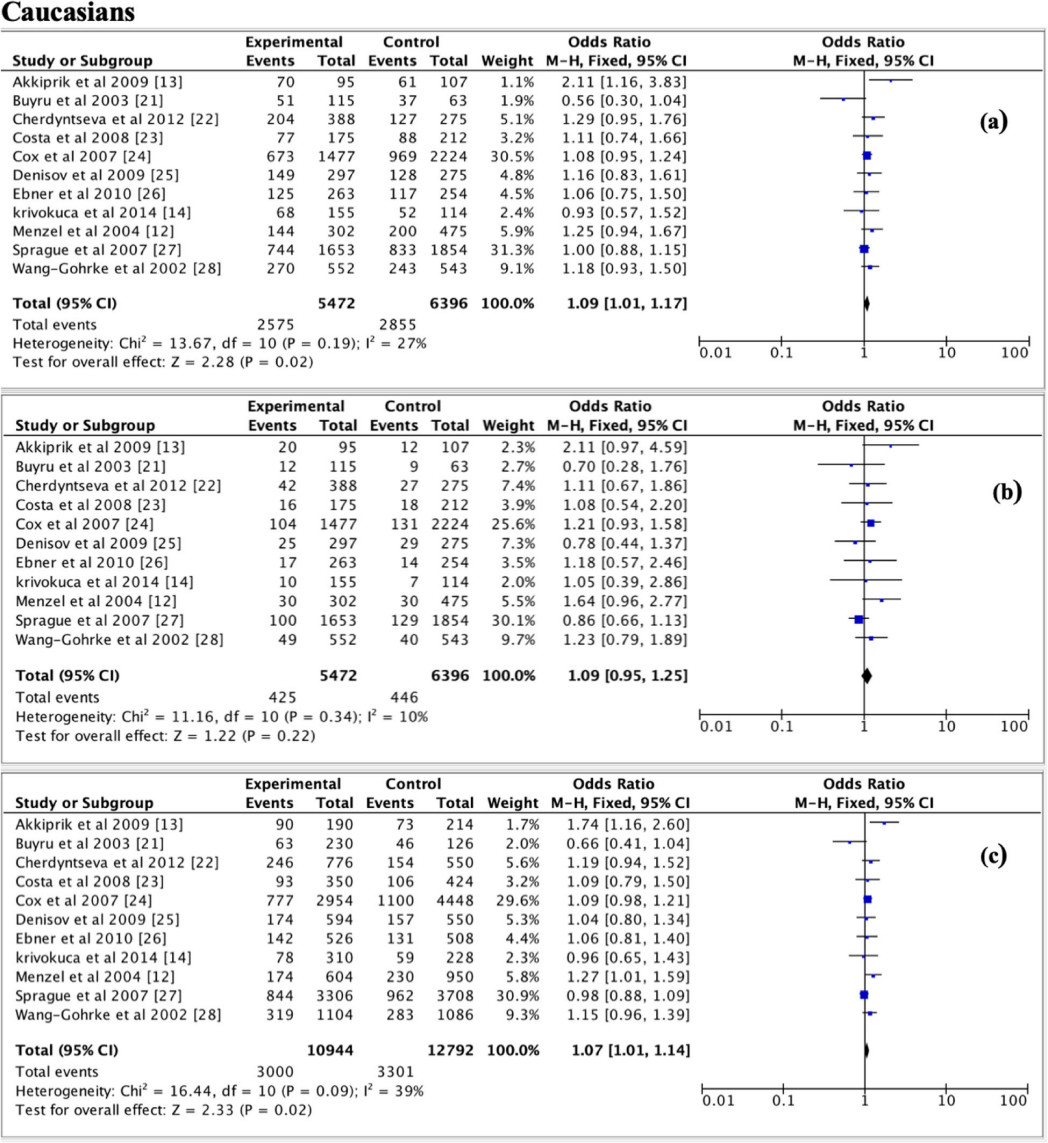
Forest plots of the association between breast cancer and *TP53* p.Arg72Pro polymorphism for the **a** dominant model, **b** recessive model and **c** additive model in Caucasians. The black diamond denotes the pooled OR; blue squares indicate the OR in each study with square sizes inversely proportional to the standard error of the OR; and horizontal lines represent the 95% CI

**Fig. 6:**
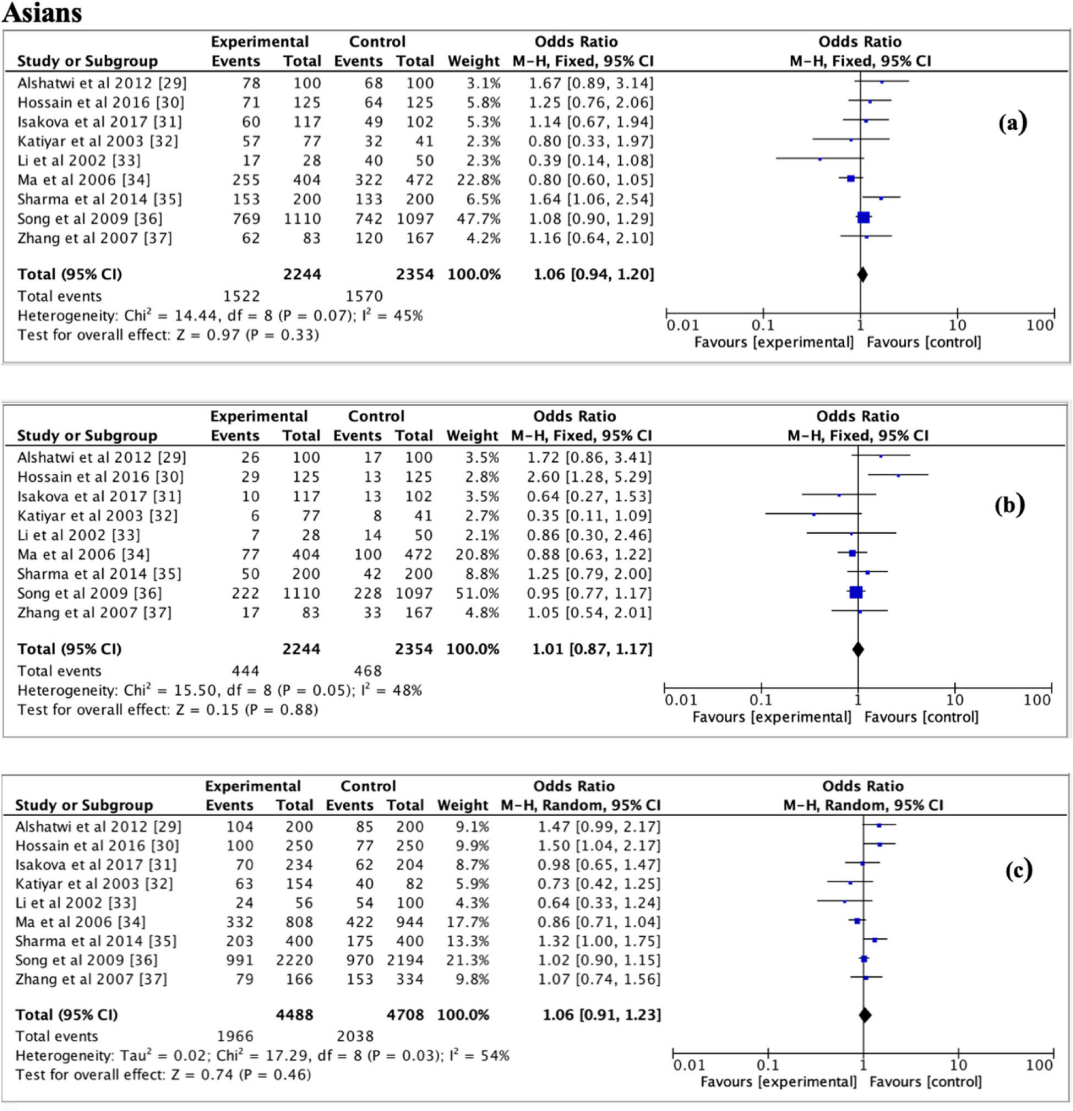
Forest plots of the association between breast cancer and *TP53* p.Arg72Pro polymorphism for the **a** dominant model, **b** recessive model and **c** additive model in Asians. The black diamond denotes the pooled OR; blue squares indicate the OR in each study with square sizes inversely proportional to the standard error of the OR; and horizontal lines represent the 95% CI

Table 2 shows pooled ORs and heterogeneity testresults of the association between the *TP53* p.Arg72Pro polymorphism and the risk of breast cancer. Overall, a slightly association of *TP53* p.Arg72Pro polymorphism with the risk of breast cancer was observed for the dominant (OR = 1.09, 95% CI = 1.02–1.16, *P* = 0.01, Fig. 2) and additive (OR = 1.09, 95% CI = 1.01–1.17, *P* = 0.03, Fig. 3) models, but not for the recessive model (OR = 1.07, 95% CI = 0.97–1.18, *P* = 0.19, Fig. 4). In the subgroup analyzes, except the recessive model (OR = 1.18, 95% CI = 0.96–1.44; *P* = 0.12), we noted a moderate association of p. Arg72Pro with the risk of breast cancer for the dominant (OR = 1.09, 95% CI = 1.01–1.17, *P* = 0.02) and additive (OR = 1.07, 95% CI = 1.01–1.14, *P* = 0.02) models in Caucasians (Fig. 5). When considering the Asian population, the different genetic models showed no trend (recessive: OR = 1.01, 95% CI = 0.87–1.17, *P* = 0.88; dominant: OR = 1.06, 95% CI = 0.94–1.20; *P* = 0.33; additive; OR = 1.06, 95% CI = 0.91–1.23, *P* = 0.46) (Fig. 6). The only eligible African study showed that the *TP53* p.Arg72Pro polymorphism is highly associated with the risk of breast cancer as well in the recessive model (OR = 2.14, 95% CI = 1.08–4.23, *P* = 0.03) than in the additive model (OR = 1.49, 95% CI = 1.03–2.16, *P* = 0.03).

**Table 2:** Distribution of *TP53* p.Arg72Pro polymorphism according to the different genetic models

		Sample size	Genetic	Pooled		Heterogeneity	
Group	N	Cases/Controls	Models	OR (95% CI)	*P*-value	I^2^	Phet
Overall	21	7841/8876					
			Dominant	1.09 (1.02–1.16) FE	0.01	31%	0.09
			Recessive	1.07 (0.97–1.18) FE	0.19	36%	0.05
			Additive	1.09 (1.01–1.17) FE	0.03	47%	0.01
Caucasian	11	5472/6396					
			Dominant	1.09 (1.01–1.17) FE	0.02	27%	0.19
			Recessive	1.09 (0.95–1.25) FE	0.22	10%	0.34
			Additive	1.07 (1.01–1.14) FE	0.02	39%	0.09
Asian	9	2244/2354					
			Dominant	1.06 (0.94–1.20) FE	0.33	45%	0.07
			Recessive	1.01 (0.87–1.17) FE	0.88	48%	0.05
			Additive	1.06 (0.91–1.23) RE	0.46	54%	0.03
African	1	125/126					
			Dominant	1.36 (0.83–2.23)	0.23		
			Recessive	2.14 (1.08–4.23)	0.03	–	–
			Additive	1.49 (1.03–2.16)	0.03	–	–

*: Significant, P: *p* value OR; I^2^: Inconsistency; dominant model*: Pro/Pro* + *Arg/Pro* vs. *Arg/Arg*; recessive model: *Pro/Pro* vs. *Arg/Arg* + *Arg/Pro*; additive model: *Pro* vs. *Arg*; Phet: *P* value of Heterogeneity, *FE* Fixed effect, *RE* Random effect, *N* Number

### Sensitive analysis

To maintain the stability of the meta-analysis after the non-inclusion of deviant studies of HWE, we evaluated the influence of each study on pooled OR. After the exclusion of studies [39–42],no study has shown a significant influence of pooled OR effect and *p*-values for the different genetic models (Table 2).

### Sources of heterogeneity

To avoid large heterogeneity, we excluded studies in which the distribution of genotypes deviated from the HWE equilibrium. The sensitivity analysis overall, showed moderate heterogeneity (I^2^ < 50%) in the recessive and dominant models. We noted the same tendency of heterogeneity when considering all the data (I^2^ = 47%, *P* = 0.01) and among Asians (I^2^ = 54%, *P* = 0.03) for the additive model (Table 2). In addition, we compared the pooled OR of the fixed and random effects, no statistically significant difference was found between the two effects, which supports strongly the consistency of the present study’s data.

### Publication Bias

**Fig. 7:**
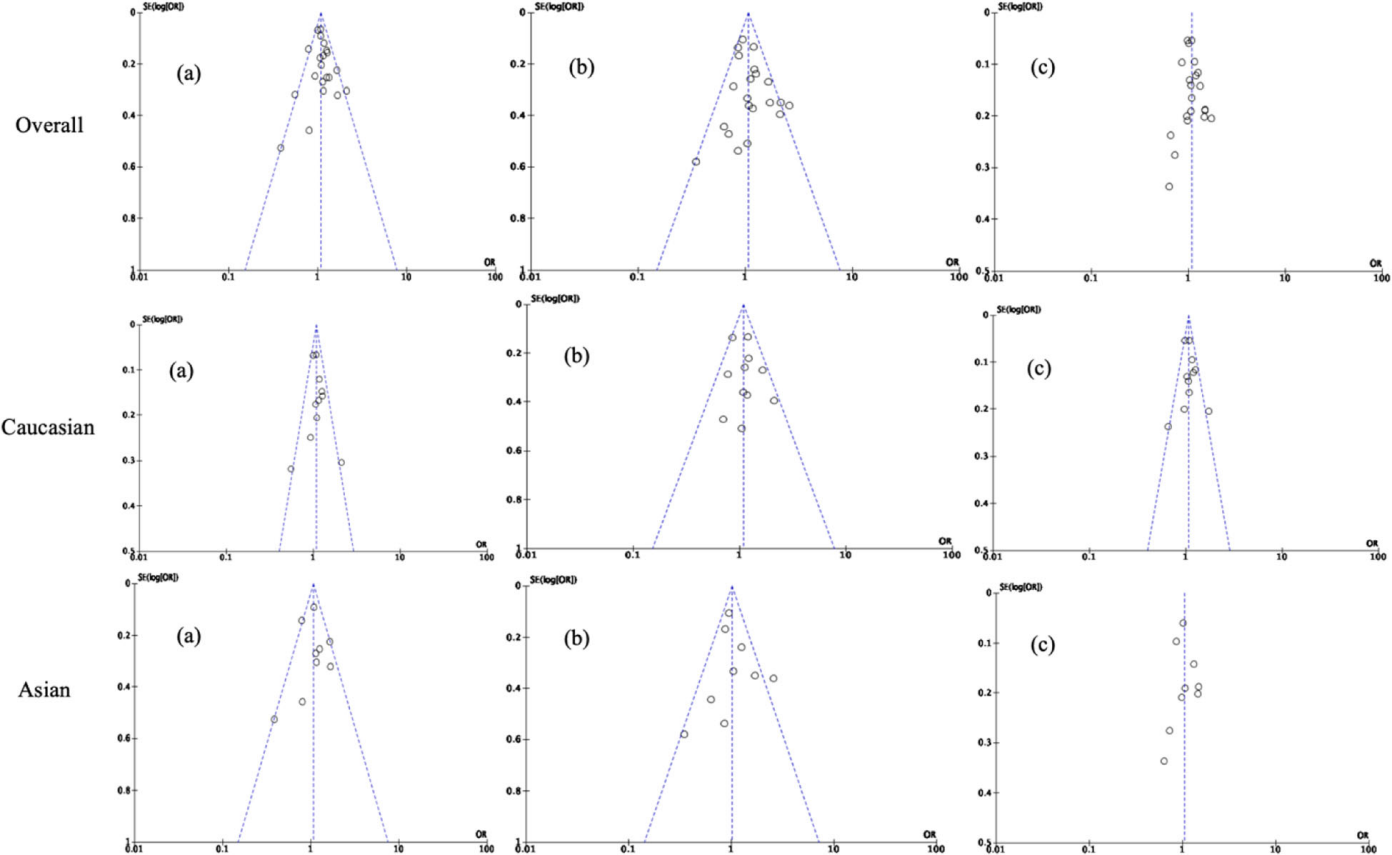
Funnel plots of dominant **a**, recessive **b** and additive **c** model precision by OR

The publication bias was assessed using the funnel plot. After the exclusion of studies deviating from HWE and those influencing the pooled ORs values, no significant publication bias was found in the different genetic models (Fig. 7).

## Discussion

Like other multifactorial diseases, the causes of breast cancer are not known. However, several factors combine their effects for the development of the disease. These factors are of clinical, biological, environmental and genetic origin [43, 44]. From a genomic point of view, it has been reported that genetic polymorphisms of the *p53* gene can influence the development of cancers [45]. However, the mechanism by which these polymorphisms affect cancer development remain unknown. Functionally, these polymorphisms alter alternative splicing and thus affecting mRNA stability and protein synthesis. The normal *P53* gene produces a protein that plays a key role in DNA repair, cell cycle control and apoptosis [45]. Through this physiological role P53 acts as a guardian of the genome, preventing the malignant transformation of normal cells. In the event of a mutation, the function of p53 is impaired, leading to the appearance of malignant cells and later cancerous disease [46–48]. It has been reported that the *R72* variant of the *P53* mutant in addition to influencing the onset of cancer is also associated with a bad prognosis through the rapid onset of metastasis [49].

In the present meta-analysis, we examined the relationship between *TP53* p.Arg72Pro polymorphism and the risk of breast cancer. Overall, our findings showed that the dominant and additive models were associated with an increased risk of breast cancer for the carriers of *72Pro* allele. These results corroborate with the data reported from two recent meta-analyzes, the first covering eleven studies with 950 cases and 882 controls in the Asian population [50] and the second performed on the Indian population which covered seven studies with 1249 cases and 1838 controls [51]. These authors in their analysis showed that the dominant and the additive models were associated with the risk of breast cancer. Contrary to our results, other meta-analyzes found conflicting results [52, 53]. The works of Zhuo et al. 2009, Francisco et al. 2011, Ma et al. 2011 and Concalves et al. 2014 also reported a decreased risk of breast cancer with the different genetic models applied [54–57]. These differences might be explained by the samples size, types of allelic variant and eligible studies included. In the subgroup analysis, our meta-analysis revealed a high risk of breast cancer with *TP53* p.Arg72Pro in Caucasians (dominant model and additive model) and Africans (recessive and additive models). These trends were consistent with previous studies [12, 13, 22] but inconsistent with the findings of other studies [52, 53]. However, Jafrin et al. 2020 concluded that *TP53* p.Arg/Pro was associated with the risk of breast cancer in the South Asian population. The difference between the studies could be explained by the ethnicity and study design. The effects of ethnicity may be due to several factors, allelic heterogeneity, gene-gene and gene-environment interaction and linkage disequilibrium [58–61]. In the previous meta-analyzes, the selection criteria of studies were not sufficiently robust such as inclusion of the age-unmatched case-control studies and the inclusion of studies with control groups not satisfying HWE [62–75]. The major advantage of the present meta-analysis was the inclusion of a large number of samples, including very selective criteria to measure the strength of the association between this polymorphism in exon 4 of *TP53* gene and the risk of breast cancer using different genetic models. However, several limitations need to be highlighted, sample size and small number of case-control age-matched studies in ethnic groups.

## Conclusion

In the light of this meta-analysis, we noticed that individuals carrying at least one *Pro* allele of the *P53* gene are more likely to have breast cancer with dominant and additive models than individuals harboring the wild-type *Arg* allele. Our study further strengthened and confirmed the hypothesis that the *P53* gene is usually mutated in about half of breast cancer cases. For the stability and homogeneity of results from meta-analysis, future similar studies should take into account selection criteria for articles such as no deviation from HWE in the control group and the matching of cases and controls according to age.

## Supplementary information

**Additional file 1.** Availability of all data and references with PubMed accession numbers.

## Data Availability

The dataset analyzed for thisstudyis available from the Additional file 1.

## Abbreviations

*Arg*: Arginine
CI: Confidence interval
Fig.: Figure
HWE: Hady-Weinberg Equilibrium
I^2^: Inconsistency
N: Number
OR: Odd ratio
P: *P* value
*Pro*: Proline
SNP: Single nucleotide polymorphisms
