# Supplementary material for: p.Arg72Pro polymorphism of P53 and breast cancer risk: a meta-analysis of case-control studies
Source: BMC Med Genet. 2020 Oct 19;21:206. doi: 10.1186/s12881-020-01133-8 (PMC7574232; doi:10.1186/s12881-020-01133-8)
Supplement: Supplementary file 1 — Additional file 1. Availability of all data and references with PubMed accession numbers. [file 12881_2020_1133_MOESM1_ESM.docx]

**Additional file 1.** Availability of all data and references with PubMed accession numbers

| **Authors** | **Accession (PubMed)** |
| --- | --- |
| Akkiprik et al 2009 [13] | PMID: 19048399 |
| Alshatwi et al 2012 [21] | PMID: 21477265 |
| Ayoubi et al 2018 [22] | PMID: 29949804 |
| Buyru et al 2003 [23] | PMID: 12684648 |
| Cherdyntseva et al 2012 [24] | PMID: 21838531 |
| Costa et al 2008 [25] | PMID: 18230179 |
| Cox et al 2007 [26] | PMID: 17387621 |
| Denisov et al 2009 [27] | PMID: 19596263 |
| Ebner et al 2010 [28] | PMID: 20127253 |
| Hossain et al 2016 [29] | PMID: 27837441 |
| Isakova et al 2017 [30] | PMID: 29132330 |
| Katiyar et al 2003 [31] | PMID: 14577584 |
| krivokuca et al 2014 [14] | PMID: 24114315 |
| Li et al 2002 [32] | PMID: 12467072 |
| Ma et al 2006 [33] | PMID: 16314399 |
| Menzel et al 2004 [12] | PMID: 15138483 |
| Sharma et al 2014 [34] | PMID: 25169539 |
| Song et al 2009 [35] | PMID: 19789321 |
| Sprague et al 2007 [36] | PMID: 17449902 |
| Wang-Gohrke et al 2002 [37] | PMID: 11927843 |
| Zhang et al 2007 [38] | PMID: 18067229 |
